# Observations on the Internal Use of Lead

**Published:** 1802-09-01

**Authors:** 

**Affiliations:** Kilkenny


### *Observations on the internal Use of Lead; communicated by Dr.* Ryan, *of Kilkenny.*

In confequence of the Controverfy that has arifen on the nature of Apoplexy, between Dr. Langflow and Mr. Crowfoot, the attention of medical practitioners has been particularly directed to the pathology and cure of this diforder. Without any intention of entering the lifts as a champion for either, I mean to confine myfelf to the hiftory and treatment of an inftance of this complaint, attended with very peculiar circumftances; and if you think it deferving a place in the Medical and Phyfical Journal, you will oblige me by inferting it.

Before I enter into a detail of this cafe, I muft beg leave to mention that I took an opportunity, not long fince, of tranfmitting you fome Obfervations on the ufe of acetite of lead in hæmorrhagy of the lungs. In a note of the Editors, in reference to the concluding fentence of this paper, they affirm that this remedy has been freely made ufe of in London ever fince Dr. Reynolds's Differtation on that fubject firft made its appearance. Though the paffage alluded to is very ftrongly worded, I neverthelefs thought myfelf, in fome meafure, juftified in making ufe of it, as from every recent publication that has come to my hands, I have not been able to difcover a fingle cafe in which the acetite of lead has been adminiftered with boldnefs or freedom; on the contrary, in all the periodical works, and in thofe of a different defcription that have noticed it, the idea of confiderable danger from its deleterious powers in very limited quantities is ftrongly inculcated. One of the laft Effays on its internal ufe that I have feen is by Mr. Clutterbuck. He gives the minutes of a convulfive diforder treated at the Univerfal Difpenfary,* in which one fourth of a grain only of this metallic falt was taken four times a day; and yet he afferts, that this inconfiderable portion was attended with very unpleafant confequences. If other practitioners are to direct their practice by this example from the capital, very little encouragement is held out to them to imitate it, as it is not eafy to comprehend how any complaint of magnitude or danger fhould readily yield to fo inferior and reduced a dofe of this medicine. Moreover, the venerable Dr. Heberden, in his eftimable pofthumous Work, enjoins phyficians never to make ufe of it from the experience he has had of its bringing on colica pictonum.

---

* Vide Medical and Phyfical Journal, Vol. IV. page 287.

A lady

A lady of high rank in this town, ætat. 61, of a thin and rather meagre habit of body, fuddenly fell fpeechlefs to the ground the latter end of the fummer, 1796. Medical aid was immediately procured, a blifter was applied between the fhoulders, one to each temple, and the bowels were emptied by a glyfter. I faw her in lefs than an hour after thefe means had been ufed, and found her labouring under a difeafe called by phyficians epileptic apoplexy.*

She had juft got out of a ftrong convulfive fit, and lay completely apoplectic, fnoring fo loudly that fhe could be diftinctly heard from an adjoining apartment. Her pulfe was unufually ftrong, and ranged from 110 to 115 ftrokes in a minute. On confidering the nature of the cafe, I had no hefitation in recommending blood-letting as the only poffible method by which her life could be preferved. She was accordingly bled to the amount of about fourteen ounces, and two leeches were applied to each temple. I faw her in about an hour after, when fhe fhewed every fymptom of returning fenfibility, and in lefs than two hours more fhe had completely thrown off the attack; but during the courfe of the night the diforder returned in the fame fhape and form as at firft, and fhe was refcued from it by a repetition of the bleeding. In both inftances the blood threw up a large portion of coagulating lymph, and had every appearance of being highly inflammatory. From this period fhe enjoyed very tolerable health for the fpace of fix months, when fhe was fuddenly feized with threatening fymptoms of a frefh attack, her fpeech fuddenly failing her fo as to render her incapable of articulating more than one or two words of one fyllable, though fhe was perfectly confcious of her fituation, and ftretched out her arm for the purpofe of having herfelf blooded, as fhe underftood her life was preferved by it in her former illnefs. Her countenance and manner were highly expreffive of the agitation of her mind, and clearly evinced how much fhe muft have been alive to the critical and impending danger fhe was in. The pulfe was equally full, quick, and vibratory as in the firft paroxyfm of her diforder. The moment affiftance could be had, fhe was copioufly blooded, and it was highly interefting to obferve how calm and tranquil fhe became during the flow of the blood; in fact, it acted more like a charm than like the ufual efficacy of remedial agents, completely

---

* It is probable fome objection may be made to this cafe as not being one in point; but as, in all its fymptoms, the complaint refembled the apoplexy more ftrongly than epilepfy, it is to be hoped its hiftory will not be confidered irrelevant to the fubject in queftion.

reftoring the power of fpeech in a few minutes, and in a very fhort time thereafter her accuftomed fpirits and vivacity.

For twelve months more no extraordinary deviation from health was obferved to occur; once or twice, indeed, fome fymptoms of beginning congeftion of blood in the head began to fhow themfelves, which were readily done away by a blifter to the neck, or by fome brifk cathartic medicine. However, fhe at length began to experience a fenfe of weight in the head, flufhings in the face, palpitation of the heart, and fpafmodic twitches in different parts of the body; thefe were confidered by herfelf, as well as by her friends, no uncertain premonitory fign of an approaching paroxyfm, and I was fent for to vifit her. While I fat by her, enquiring into the ftate of her health, her fpeech fuddenly failed, and fhe feemed to be in the utmoft horror and agony of mind imaginable. To avert, if poffible, the ftorm that every inftant was expected to burft, the apothecary who ufually attended was defired to hurry as faft as poffible; but before he had time enough to arrive and tye up the arm, fhe dropped from her chair convulfed and infenble; blood to the amount of near 14 ounces was taken from the arm by a large orifice, and the bowels were emptied by a glyfter; but no advantage having been obtained by this quantity, and the pulfe remaining as full and energetic as before the bleeding, it was determined upon to have recourfe to it a fecond time, in nearly the fame proportion, after having waited for the fpace of an hour to fee the refult of the firft. This, however, appearing as ineffectual as the former, it was thought expedient to fly to the aid of blifters; accordingly, they were applied to the whole fcalp of the head, between the fhoulders, to the legs, and finapifms were laid on the foles of the feet. She remained under the influence of thefe, without feeling or motion, upwards of four hours; and inftead of any amendment having taken place, the profpect every inftant became fo gloomy and alarming, that death it was thought muft ineviatbly have clofed the fcene in a fhort time, unlefs fomething further was attempted in a prompt and decifive manner. Though the lofs of blood already fuftained was confiderable, the pulfe ftill retained its hardnefs and frequency; the face was flufhed and bloated; and from the rapidity with which the blood was driven to the head, the pulfation of the temporal and carotid arteries was diftinctly perceptible, the moment the eye was directed to the countenance.

Under thefe circumftances, as a laft effort, the temporal artery was opened. The blood was allowed to flow till the force of the circulation was effectually fubdued. This was not accomplifhed until between twelve and fourteen ounces were

 drawn.

drawn. 'After this ſhe began to emerge from her depreſſed and almoſt lifeleſs ſituation, gradually recovering her mental and bodily powers; and in leſs than ſix hours from the laſt bleeding, ſhe had not the ſmalleſt remnant of her diſorder except what was occaſioned by the exceſſive loſs of blood and the irritation of the bliſter.

From this time to her death, which took place the latter end of December laſt, ſhe experienced but one formidable return of her complaint that menaced the exiſtence of life; it was in conſequence of a ſhock ſhe received from unexpectedly ſeeing a favourite daughter who had been long abſent, and who was the object of her love and adoration. After her arrival was announced, ſhe endeavoured to ſummon up all the reſolution ſhe poſſeſſed, in order to reſiſt, if poſſible, the forcible impreſſion ſhe well knew the ſight of her beloved child would make; but all to no purpoſe, as immediately after the interview had taken place, ſhe fell down ſpeechleſs and apoplectic, and in this hopeleſs ſtate remained for eight or ten hours, till ſhe was ſnatched from it by exactly the ſame vigorous and deciſive meaſures that were adopted in the firſt attack.

The intellectual faculties more tardily developed themſelves after this than after any former illneſs, an imbecility of mind remaining for many days after all apprehenſions about life had vaniſhed; but her conſtitution, ſhattered by the frequent attacks it endured, could not long hold out; and after languiſhing about two months, ſhe was carried off by an inflammation of her lungs, at the age of ſixty-ſeven or ſixty-eight years.

Her ſtate of health during the length of time that intervened between the ſecond and laſt attack (a ſpace of between four and five years) was fluctuating and chequered. In the winter ſeaſon, and particularly when the weather was cold, the diſorder ſhewed very little diſpoſition to recur; but in the warmer months of ſummer it was found neceſſary to have a watchful eye upon her, leſt a paroxyſm ſhould come on from the turgid ſtate of the veſſels of the brain, evidently induced by the power of heat on the arterial ſyſtem. On theſe occaſions, taking blood by opening the temporal artery, or by the application of leeches, was certain of affording inſtantaneous relief.

In this lady's caſe, a ſtriking peculiarity demands particular notice. In complete abſence of diſeaſe, and full enjoyment of health, the general ſtandard of the pulſe was about 100, and ſeldom leſs than 95 ſtrokes in a minute, though occaſionally liable to variations from accidental cauſes. The beat of the artery too was unnaturally hard, ſo as to authoriſe one to pronounce it a highly morbid ſtate of action, though all the other functions tions of the body were apparently in perfect harmony and order.

The plan of cure, or rather of prevention, was conducted on the principle of avoiding every thing that could produce excitement in the syftem, while it was equally an object in view to guard againft too great a reduction of the energy and powers of the conftitution. Animal food was allowed in moderate quantity, and white meats were chiefly recommended and made ufe of; but wine, in every fhape and form, was prohibited; the conftant beverage was lemonade, or cyder diluted with a large proportion of water. An iffue was cut in the neck, and one inferted above the knee, at each fide, in the ufual place; the head was regularly fhaved twice a week, and cold water poured on it every morning.

At one time a diet confifting of vegetable and farinaceous matter was entered upon, and perfevered in with rigour for upwards of two months, without any vifible effect in diminifhing the plethoric ftate of the veffels of the brain; on the contrary, fome very ferious appearances of plenitude began to be manifeft, and which it was found neceffary to obviate by topical blood-letting. Far different was the effect of deducting from the ordinary quantity of liquids taken in; a reduction of one-third, and fometimes one-half, of the ufual daily allowance of drink, was enjoined, and frequently attended to with ftrictnefs and punctuality. The efficacy of this was prominent and ftriking above every other remedy employed; the pulfe loft of its force, of its hardnefs, of its frequency and fullnefs. So fudden and manifeft was the tranfition from morbid to healthy action, after the adoption of this plan, that the exemption from difeafe that took place, and the prolongation of life, may be in a great meafure attributed to its influence.

